# Hints on the Obstetric Practice

**Published:** 1876-01

**Authors:** 


Hints on the Obstetric Procedure. By William B. Atkinson, M.D., Philadelphia. Book pp. 89.

This little work comes neatly bound and arranged with taste. The author claims that no attempt has been made to exhaust the subject, or offer a complete *vade mecum.* He begins the work by a description of false pains, dilatation, administration of ergot, opium, etc., rupture of membrane, a description of instrumental delivery, etc. The work closes with a few chapters on convulsions, nourishment of child and mother, etc. Upon the whole, it is an admirable little work for reference, and no doubt contains that which its author claims for it.

